# Hexa-Graphyne: A Transparent and Semimetallic 2D Carbon Allotrope with Distinct Optical Properties

**DOI:** 10.1021/acsomega.5c12156

**Published:** 2026-02-03

**Authors:** Jhionathan de Lima, Felipe Hawthorne, Cristiano F. Woellner

**Affiliations:** † Department of Physics, 28122 Federal University of Parana, UFPR, Curitiba, PR 81531-980, Brazil; ‡ Interdisciplinary Center for Science, Technology, and Innovation (CICTI), Federal University of Parana, UFPR, Curitiba, PR 81530-000, Brazil

## Abstract

Herein, we conduct a comprehensive investigation of Hexa-graphyne (HXGY), a planar carbon allotrope formed by distorted hexagonal and rectangular rings incorporating sp and sp^2^-hybridized carbon atoms. First-principles calculations confirm its energetic, dynamical and thermal stability (up to at least 1000 K). Regarding its band structure, this material exhibits a semimetallic nature. It exhibits high mechanical compliance, with a Young’s modulus approximately 13 times lower and a Poisson’s ratio nearly 4 times higher than those of graphene. The optical response is marked by strong ultraviolet absorption, high infrared reflectivity, and pronounced transparency in the visible-light range. Raman and infrared spectra exhibit sharp and well-separated peaks, providing a clear signature of acetylenic linkage stretching vibrations. Nanoribbon structures derived from HXGY show distinct electronic behaviors depending on the edge termination type and width. These findings highlight the HXGY potential for nanoelectronic and optoelectronic applications.

## Graphical abstract

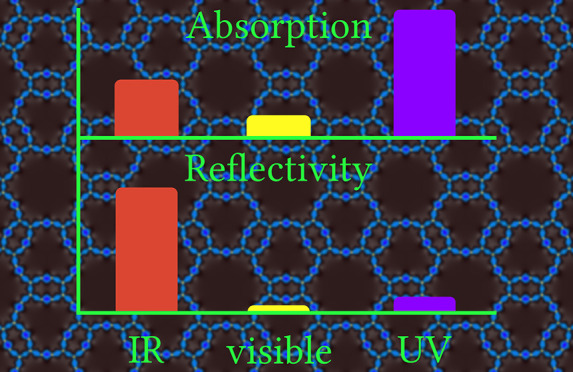

## Introduction

The structural diversity of carbon allotropes stems from the ability of this element to form three distinct covalent bonds via sp, sp^2^, and sp^3^ hybridizations. In nature, carbon can be found primarily as diamond, a three-dimensional (3D) network of sp^3^-hybridized atoms, and graphite, composed of stacked layers of sp^2^-hybridized atoms.

In the last decades, significant efforts have been dedicated to synthesize new carbon allotropes, and a considerable progress has been achieved. The most notable examples are zero-dimensional (0D) fullerenes,^1^ one-dimensional (1D) carbon nanotubes,^2^ and two-dimensional (2D) graphene.^3^ In particular, graphene stands out as a material of unique interest, owing to its remarkable physicochemical properties.^4^ However, the gapless nature of graphene has motivated extensive research on specific physical and/or chemical modifications aiming to open a band gap in its electronic signature.^5,6^

While the electronic properties of graphene can be partially tuned by cutting it into 1D nanoribbons,^7,8^ experimental control over edge terminations (zigzag or armchair) in such structures remains a challenge. Approaches such as bottom-up synthesis or postgrowth chemical functionalization are often required to stabilize the edges and preserve their intrinsic electronic properties.^9–11^

An alternative research pathway focuses on designing 2D carbon allotropes with structural configurations fundamentally distinct from graphene. A prominent approach involves incorporating sp-hybridized carbon atoms via acetylenic groups (−C≡C−) into a graphitic network, giving rise to the graphyne (GY) family.^12^ These structures are typically defined relative to the graphene lattice and differ primarily in the atomic density, spatial distribution and length of acetylenic groups across the lattice.^13,14^ Among the known GY structures, α-, β-, and γ-types have been the most extensively studied.^15–17^ Although most members of the GY family remain theoretical predictions, the successful synthesis of a few representatives variants such as γ-GY^18–22^ and γ-GDY,^23^ has driven this research direction, underscoring the predictive power of computational studies.

Despite these advances, the interplay between structural arrangement, bond hybridization, and pore architecture suggests a vast design space where new GYs may exhibit exceptional optoelectronic properties not present in graphene or in the known α-, β-, and γ-forms. A recent trend in the literature is the design of GYs based on nongraphitic, full-sp^2^ carbon lattices. Notable examples include GYs inspired in the Biphenylene network,^24,25^ and Pentagraphene.^26^ In a recent contribution to this field, Mavrinskii and Belenkov proposed seven new polymorphs of GYs layers derived from the Graphenylene.^27^ Their work established initial insights into the relative stability of these new structures via sublimation energies, providing a preliminary analysis of their electronic band structures. However, it primarily focused on structural classification and comparative energetics, leaving the stability evaluation and key physical properties such as mechanical, optical and vibrational properties of these systems unexamined. These elements are crucial to validate the feasibility of these 2D carbon allotropes for experimental realization and potential applications. A subsequent study has confirmed the dynamical stability and semiconducting character of one member of this family,^28^ demonstrating the potential for further investigation of these structures.

Building on this progress, the present work provides a comprehensive first-principles investigation of the structure designated as β2 – *L*_4–6–12_ in the original study by Mavrinskii and Belenkov. For the sake of simplicity, we will refer to it as Hexa-graphyne (HXGY) throughout this paper. HXGY is composed of sp- and sp^2^-hybridized carbon atoms forming distorted hexagonal rings interconnected by rectangular units, arranged in a hexagonal lattice. Using all-electron first-principles calculations based on density functional theory (DFT), we conduct a comprehensive analysis and characterization of its stability and fundamental properties. We further investigate the electronic properties of 1D nanoribbons derived from the HXGY lattice, elucidating how edge termination and width modify the electronic structure relative to the parent 2D material.

## Methodology

We performed electronic structure calculations using the all-electron Fritz Haber Institute Ab-Initio Molecular Simulations (FHI-AIMS)^29^ code. Within this framework, the electron density and all the operators are expand over a numerical-atomic-orbitals basis set. The predefined “tight” basis set option was employed for all elements to ensure higher accuracy. Exchange-correlation effects were treated with the Perdew–Burke–Ernzerhof (PBE) functional within the general gradient approximation (GGA).^30^ The hybrid Heyd–Scuseria–Ernzerhof (HSE06)^31^ functional was also used to obtain an accurate band gap and light absorption properties.

The optimized structures were obtained by relaxing the atomic positions and lattice vectors until the maximum force on each atom was below 10^–3^ eV/Å, and the total energy difference was less than 10^–6^ eV. Structural optimization and static electronic calculations were performed using a Monkhorst–Pack *k*-point mesh of 32 × 32 × 1. A denser 64 × 64 × 1 mesh was employed for density of states (DOS) calculations. To eliminate spurious interactions between periodic images, a vacuum layer of 20 Å was added along the out-of-plane direction. For the nanoribbon models, periodic boundary conditions were applied along the ribbon axis, with at least 20 Å for the vacuum buffer layer along the nonperiodic directions. The corresponding Brillouin zones were sampled with a 1 × 8 × 1 *k*-point mesh.

The cohesive (*E*_coh_) and formation (*E*_form_) energies were computed using the relations *E*_coh_ = (*E*_total_ – *NE*_C_)/*N* and *E*_form_ = (*E*_total_ – *N E*_graphene_)/*N*, respectively. In these equations, *E*_total_ is the total energy of HXGY, *E*_C_ is the energy of an isolated carbon atom, *E*_graphene_ is the energy per atom of graphene, and *N* is the total number of carbon atoms in the unit cell.

Phonon dispersion calculations were carried out for a 2 × 2 × 1 supercell applying density functional perturbation theory (DFPT) as implemented in the Phonopy package.^32^ Additionally, *ab initio* molecular dynamics (AIMD) simulations were conducted in the NVT ensemble using a Nosé-Hoover thermostat^33,34^ for temperature control. These simulations were run at temperatures of 300 and 1000 K for a duration of 5 ps with a 1 fs time step. For this purpose, we used the i-PI code^35^ to manage the molecular dynamics, while FHI-AIMS computed the forces on-the-fly.

Elastic constants (*C*_*ij*_) were calculated as the second derivative of the energy (*E*) with respect to strain components (ε_*i*_ and ε_*j*_) according to the expression $C_{\mathrm{ij}}=\frac{1}{A_{0}}\frac{\partial^{2}E}{\partial \varepsilon_{i}\partial \varepsilon_{j}}$ , where *A*_0_ is the surface area of the unstrained unit cell.

Optical properties were evaluated within the random phase approximation (RPA) framework^36^ using the frequency-dependent complex dielectric function ϵ­(ω) = ϵ_1_(ω) + *i* ϵ_2_(ω), where ω is the photon energy. The imaginary part ϵ_2_(ω) was obtained directly from interband transitions, while the corresponding real part ϵ_1_(ω) was derived via the Kramers–Kronig transformation.^37^ Once the real and imaginary parts of the dielectric function are determined, it becomes possible to calculate key optical coefficients, including the absorption coefficient

1 $$\alpha \left(\omega \right)=\sqrt{2}\omega {\left[\sqrt{\epsilon_{1}^{2}+\epsilon_{2}^{2}}-\epsilon_{1}\right]}^{1/2}$$

the refractive index

2 $$n\left(\omega \right)=\frac{\sqrt{2}}{2}{\left[\sqrt{\epsilon_{1}^{2}+\epsilon_{2}^{2}}+\epsilon_{1}\right]}^{1/2}$$

and the reflectivity

3 $$R\left(\omega \right)={\left|\frac{\sqrt{\epsilon_{1}+i\epsilon_{2}}-1}{\sqrt{\epsilon_{1}+i\epsilon_{2}}+1}\right|}^{2}$$

## Results and Discussion

### Structural Properties

The optimized structure of HXGY, shown in Figure 1a, exhibits a hexagonal unit cell composed of 36 carbon atoms, belonging to the *P*6/*mmm* space group (No. 191). The optimized lattice parameters are *a* = *b* = 14.05 Å, with angles α = β = 90 ° and γ = 120 °. These structure is composed of distorted hexagonal rings fully edged by acetylenic groups, which are interconnected through distorted rectangular rings. These rectangular units connect hexagons by sharing acetylenic sides and bridging them via sp^2^-hybridized carbon atoms. The periodic repetition of the unit cell leads to the formation of extended dodecagonal large-ring motifs with an effective diameter of approximately 8.66 Å. The presence of such large pores suggests promising applications in gas storage and separation, energy storage, and water purification.

**Figure 1:**
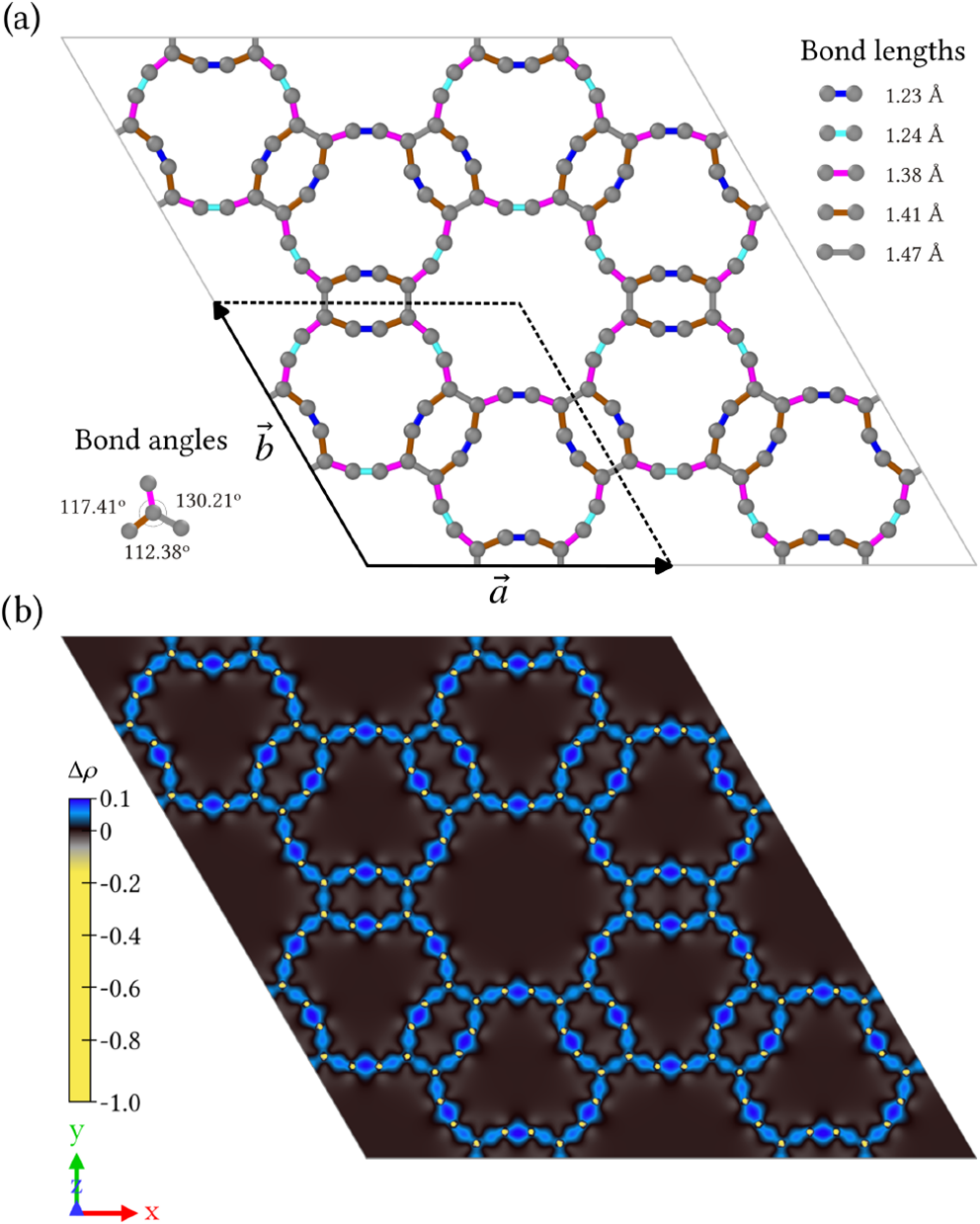
(a) Optimized atomic structure of HXGY, showing the different C—C bond lengths and bond angles. The hexagonal unit cell and lattice vectors are indicated by the black box. (b) Charge density difference (Δ*ρ*) between the self-consistent electronic density and the superposition of isolated atomic densities. Positive values (yellow) indicate charge accumulation in interatomic regions associated with covalent bonding, while negative values (blue) correspond to charge depletion around the atomic cores. The isosurface level is set to ± 0.003 e Å^–3^.

The unique structural framework of HXGY leads to nonuniform bond lengths and bond angles. As highlighted in Figure 1a, bond lengths vary from 1.23 to 1.47 Å, and bond angles at the vertices range from 112.38 to 130.21°. These bond distances follow the expected trend according to bond order, resulting from the mixed hybridization. For example, the shortest distances (1.23 and 1.24 Å) correspond to bonds between sp-hybridized carbon atoms, where higher charge accumulation occurs. These values are comparable to those in the acetylene ground state (1.21 Å^38^). Notably, these acetylenic linkers are also slightly distorted from a linear geometry. The bond lengths between sp- and sp^2^-hybridized atoms are 1.38 and 1.41 Å, consistent with an intermediate bond order between 2 and 3. Finally, the largest bond length of 1.47 Å corresponds to bonds between sp^2^-carbon atoms and is similar to that in graphene (1.42 Å^39^).

To further elucidate the electronic origin of the bonding diversity present in HXGY, we computed the charge density difference (Δ*ρ*) between the self-consistent electronic density and the superposition of isolated atomic densities. As illustrated in Figure 1b, positive values are concentrated in interatomic regions, indicative of covalent bonding and charge accumulation. This accumulation is particularly strong and localized along the acetylenic linkages, followed by the terminal C^sp^ – C^sp^2^^ bonds and the C^sp^2^^ – C^sp^2^^ bridges. Negative values, localized around the atomic cores, correspond to charge depletion. This charge redistribution pattern clearly highlights the coexistence of both sp- and sp^2^-hybridized carbon atoms in the HXGY monolayer, and directly explains the observed variations in bond lengths and angles.

### Structural Stabilities

#### Energetic Stability

To assess the energetic stability of HXGY and place it within the broader landscape of two-dimensional carbon allotropes, we evaluated its cohesive and formation energies and compared them with representative carbon-based systems. The cohesive energy of HXGY is found to be −8.24 eV/atom, and the negative value indicates intrinsic thermodynamic stability. This value is comparable to those of other known carbon allotropes, such as graphene (−9.24 eV/atom), γ-GY (−8.60 eV/atom), γ-GDY (−8.47 eV/atom), and is close to that of β-GY (−8.40 eV/atom) and α-GY (−8.31 eV/atom), all calculated within the present study. HXGY is less stable than α-GY, which also contains hexagonal rings. This difference can be attributed to the angular strain introduced by the rectangular units in HXGY, resulting in distorted hexagonal motifs.

We further computed the formation energy of HXGY (1.00 eV/atom) to contextualize its thermodynamic cost of formation relative to other sp/sp^2^ carbon allotropes. This value is close to those of experimentally realized γ-GY (0.64 eV/atom) and γ-GDY (0.76 eV/atom), as well as to α-BPNGY^25^ (0.96 eV/atom) and α-GPGY^28^ (0.98 eV/atom), placing HXGY within a class of comparatively high-formation-energy two-dimensional carbon allotropes.

It should be emphasized that formation energy comparisons are used here solely to establish a thermodynamic reference and should not be interpreted as evidence of kinetic feasibility or direct synthetic accessibility, which would require explicit analysis of reaction pathways and transition-state barriers. Accordingly, while HXGY is energetically less favorable than graphene (used as reference), largely due to the presence of triple bonds, its formation energy situates it within an energetic regime comparable to other graphyne-based systems that have motivated experimental exploration.

From an experimental perspective, the realization of HXGY could plausibly follow bottom-up synthesis strategies similar to those successfully employed for γ-GY and γ-GDY. In particular, recent experimental breakthroughs have demonstrated the feasibility of extended sp/sp^2^ carbon networks through mechanochemical routes^18^ and scalable synthesis of multilayer γ-graphyne.^1,21^ Additionally, comprehensive reviews of both theory and experiments on graphdiyne highlight the substantial progress in synthesizing, characterizing, and applying this class of sp/sp^2^ carbon allotropes.^40^ Surface-assisted bottom-up approaches based on tailored acetylenic precursors on noble-metal substrates have also proven effective for constructing atomically precise carbon nanostructures,^9^ and related strategies have enabled the synthesis of graphdiyne thin films^23^ and the transformation of γ-GY into planar sp^2^ phases.^22^ While the specific precursor chemistry required for HXGY remains to be identified, its structural similarity to experimentally realized graphyne- and graphdiyne-based systems suggests that its synthesis may be achievable with further advances in molecular design and surface-assisted reactions.

#### Dynamic and Thermal Stability

The dynamical stability of HXGY was verified by computing its phonon band dispersion along the high-symmetry paths of the Brillouin zone, as shown in Figure 2. The absence of imaginary modes (which would conventionally appear as negative frequencies) across the entire spectrum indicates that the material is dynamically stable. As expected for a 2D material, the phonon dispersion of HXGY exhibits three acoustic branches originating at the Γ-point. Most phonon branches are observed at low frequencies (below 20 THz). The dispersion is reduced in the 25 to 45 THz range. After a forbidden region, a set of isolated bands is observed in the vicinity of 60 THz. These modes are related to the vibrations of the sp atoms in the acetylenic groups, as evidenced by the phonon projected density of states (right panel of Figure 2), and are commonly found in GY-like systems.^41^

**Figure 2:**
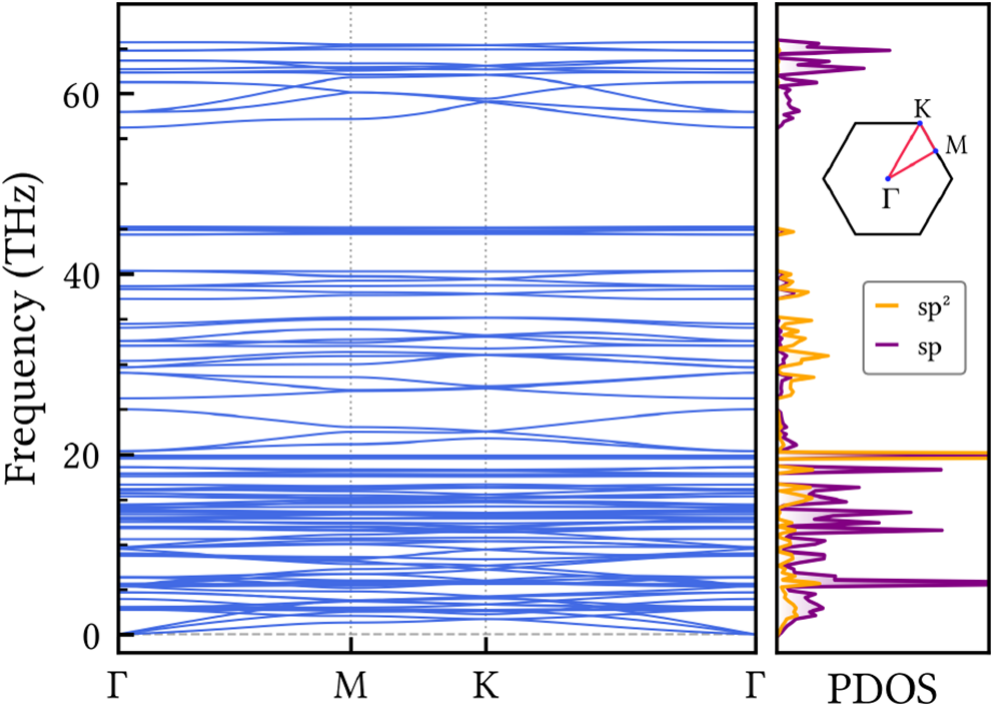
Phonon band structure and projected density of states (PDOS) of HXGY along high-symmetry lines in the Brillouin zone, with the corresponding path labeled in the inset. The dynamical stability of the system is confirmed by the absence of complex frequencies (which would conventionally appear as negative frequencies in the plot).

The thermal stability of HXGY was evaluated through AIMD simulations at finite initial temperatures of 300 and 1000 K. During the entire simulated dynamics, no bond breaking or formation was observed from a visual inspection of the trajectory. Beyond visual inspection of the simulation, Figure 3 shows the temporal evolution of the total energy, which exhibits only minor fluctuations around a steady level at all temperatures, indicating structural robustness. The corresponding final snapshots of the atomic configurations for both temperatures, presented in the insets, further corroborates this observation. The top and side views reveal the planar integrity preservation of HXGY, with only minor out-of-plane distortions emerging. In addition, the average temperatures shown in the legend of Figure 3 deviate by less than 2% from the target value, reflecting an excellent energy–temperature balance throughout the simulation. These results confirm that HXGY maintains robust thermal stability up to at least 1000 K.

**Figure 3:**
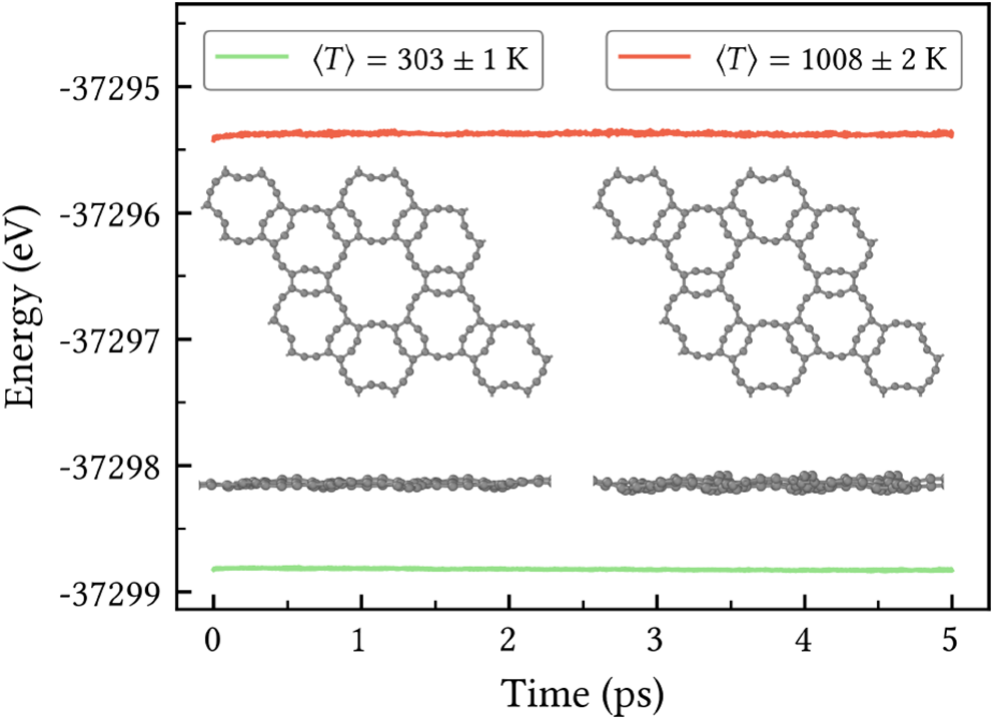
Total energy time evolution during AIMD simulations at initial temperatures of 300 K (green) and 1000 K (orange). The legend indicates the average temperatures for each simulation. The insets show the top and side views of the final atomic configurations.

#### Mechanical Stability

The mechanical stability of HXGY was assessed by computing the elastic tensor components according to the energy-strain method. This approach involves applying a set of small, finite deformations to the equilibrium lattice parameters and calculating the resulting change in total energy. The elastic strain energy *U*(ε), defined as the difference between the total energy of the strained and unstrained systems per unit area, is related with the strain components according to the following relation

4 $$U\left(\varepsilon \right)=\frac{1}{2}C_{11}\varepsilon_{\mathrm{xx}}^{2}+\frac{1}{2}C_{22}\varepsilon_{\mathrm{yy}}^{2}+C_{12}\varepsilon_{\mathrm{xx}}\varepsilon_{\mathrm{yy}}+2C_{66}\varepsilon_{\mathrm{xy}}^{2}$$

In this equation, *C*_11_, *C*_22_, *C*_12_, and *C*_66_ are the components of the stiffness tensor, corresponding to 1 – *xx*, 2 – *yy*, and 6 – *xy* according to the standard Voigt notation.^42^ For systems organized in hexagonal lattices, this general relaxation between energy and strain is further simplified. This reduction arises from the symmetry-imposed conditions *C*_11_ = *C*_22_ and the Cauchy relation 2*C*_66_ = *C*_11_ – *C*_12_, leading to the following expression:^43^

5 $$U\left(\varepsilon \right)=\frac{1}{2}C_{11}\left(\varepsilon_{\mathrm{xx}}^{2}+\varepsilon_{\mathrm{yy}}^{2}+2\varepsilon_{\mathrm{xy}}^{2}\right)+C_{12}\left(\varepsilon_{\mathrm{xx}}\varepsilon_{\mathrm{yy}}-\varepsilon_{\mathrm{xy}}^{2}\right)$$

The *C*_*ij*_ coefficients are then obtained by fitting the energy variation to a polynomial function of the strain. The uniaxial strain yields ε_*xy*_ = ε_*yy*_ = 0, which reduces the energy-strain relation to $U\left(\varepsilon \right)=\frac{1}{2}C_{11}\varepsilon_{\mathrm{xx}}^{2}$ . Parabolic fitting of this curve gives the value of the elastic constant *C*_11_. Under equi-biaxial strain (ε_*xx*_ = ε_*yy*_) the relation becomes *U*(ε) = (*C*_11_ + *C*_12_) ε_*xx*_^2^. Fitting this curve yields the sum *C*_11_ + *C*_12_, allowing *C*_12_ to be determined by substituting *C*_11_. Finally, *C*_66_ is computed as *C*_66_ = (*C*_11_ – *C*_12_)/2.

Applying the aforementioned procedure to the data in Figure 4 yields the following elastic constants for HXGY, *C*_11_ = 54.48 N/m, *C*_12_ = 39.54 N/m, and *C*_66_ = 7.47 N/m. These values obey the Born-Huang criteria^44^ for hexagonal lattices, *C*_11_ > |*C*_12_| and *C*_66_ > 0, predicting HXGY as a mechanically stable material.

**Figure 4:**
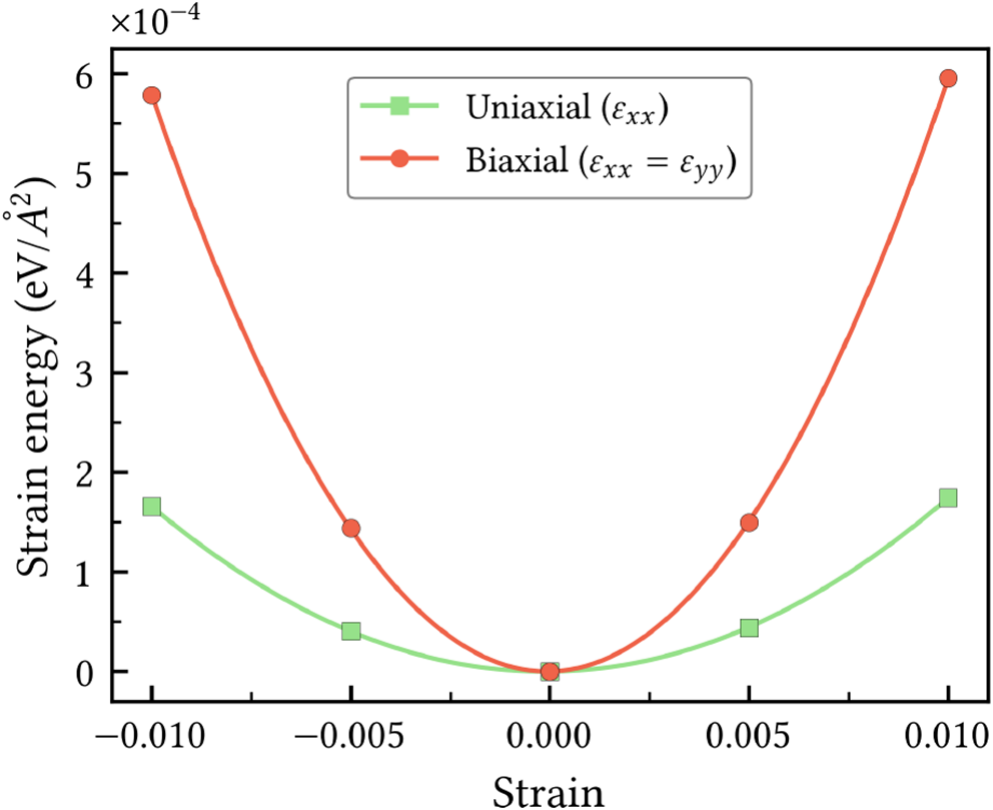
Variation of the elastic strain energy as a function of uniaxial and biaxial strain applied to the HXGY lattice vectors.

### Mechanical Properties

Following the assessment of mechanical stability, the mechanical properties of HXGY were derived from the elastic tensor components, and afterward compared with other hexagonal 2D materials. The in-plane Young’s modulus (*Y*) has been evaluated using the relation *Y* = (*C*_11_^2^ – *C*_12_^2^)/*C*_11_, and is found to be *Y* = 25.78 N/m. This value is lower than that for graphene (342.17 N/m), γ-GY (166.12 N/m), and γ-GDY (123.21 N/m), and comparable to that of α-GY (22.70 N/m), all calculated in the present study. Thus, HXGY is a very soft material, which reflects the effect of its porous structure and extended acetylenic chains, which allow the lattice to deform more readily under applied stress.

The Poisson’s ratio (ν) has been computed as ν = *C*_12_/*C*_11_, and is found to be ν = 0.73. This result is comparable to that of β-GY (0.67) and it is approximately four times greater than that of graphene (0.18), all determined in the present analysis. It is worth to note that perfectly incompressible material has Poisson’s ratio of 0.5 and more the deviation from this value toward zero implies more compressible the system is. The large ν of HXGY can be attributed to its unique geometry, where slightly curved acetylenic bridges (see Figure 1a) in the hexagonal rings accommodate deformation primarily through bond-angle bending rather than bond stretching.

The high flexibility of HXGY is consistent with its low areal density of 0.21 atom/Å^2^, defined as the total number of carbon atoms per unit area of the unit cell. This value is much lower than that of graphene (0.38 atom/Å^2^), and comparable to that of α-GY (0.19 atom/Å^2^) and β-GY (0.23 atom/Å^2^), all determined herein. This structural sparsity means significantly fewer carbon bonds per unit area can be elongated or compressed under stress, leading to the observed low in-plane elastic constants of HXGY.

### Electronic Properties

#### 2D Monolayers

In Figure 5a we present the band structure of HXGY along the high symmetry lines of the Brillouin zone, as well as the corresponding projected density of states (PDOS) for sp- and sp^2^-hybridized carbon atoms. A notable observation is the maxima of valence band (VB) and minima of conduction band (CB) approaching each other at two different off-symmetry points along the Γ → M and K → Γ integration paths. Ultimately, these bands touch at the Fermi level, resulting in a nonlinear dispersion with a gapless, semimetallic character, in agreement with the results.^27^

**Figure 5:**
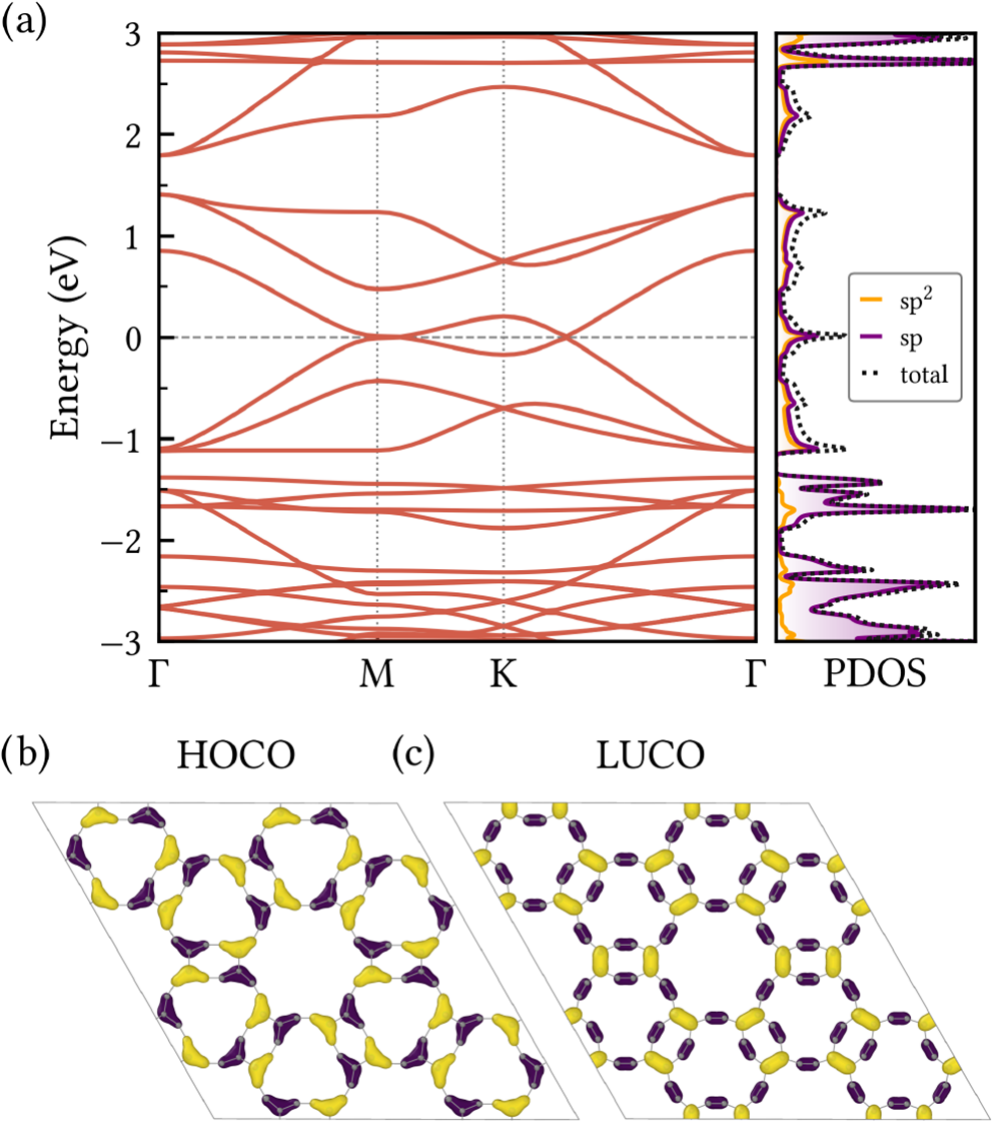
(a) Electronic band structure and projected density of states (PDOS) of HXGY calculated at the PBE level. The horizontal gray dashed line indicates the Fermi level. Visual representations of (b) the highest occupied crystalline orbital (HOCO) and (c) the lowest unoccupied crystalline orbital (LUCO), with yellow and purple colors denoting different orbital phases.

A closer examination of the PDOS reveals similar contributions from sp- and sp^2^-hybridized carbon atoms within the energy window of 1.3 eV around the Fermi level. Naturally, the sp contribution is slightly larger than the sp^2^ one in this region, as we have two sp-hybridized carbon atoms for each sp^2^ one in the HXGY structure. We also note that HXGY presents two forbidden energy regions in its band structure, one below the three highest VBs and another above the three lowest CBs. Such features could be explored in electronic transport configurations, where they may enable effects as negative differential resistance.^45^

The spatial distribution of the frontier electronic states is presented at the bottom of Figure 5, with the highest occupied crystalline orbital (HOCO) shown in panel (a) and the lowest unoccupied crystalline orbital (LUCO) depicted in panel (b). They correspond to band-edge Bloch states of the periodic lattice and are shown here to visualize the real-space character of the valence- and conduction-band edges. Both states extend across the lattice, indicating electronic delocalization and corroborating the gapless character of the system. In the HOCO, the charge density is predominantly concentrated along the C^sp^ – C^sp2^ – C^sp^ bridges at the ring vertices, oriented outward from the rectangular rings. On the other hand, LUCO extends over both C^sp^≡C^sp^ and C^sp2^ – C^sp2^ bonds, revealing a broader spatial distribution that could enable isotropic or multidirectional electron conduction. These complementary patterns between the occupied and unoccupied frontier crystalline orbitals suggests an intrinsic electronic anisotropy in HXGY. Such anisotropy is highly desirable for direction-selective device applications, including field-effect transistors and anisotropic optoelectronic platforms.

Recognizing the known limitations of GGA functionals in predicting precise band gap values, we also calculated the electronic band structure of HXGY using the HSE06 hybrid functional, as illustrated in Figure 6. Compared to the PBE/GGA results, the primary difference is a systematic outward shift of the bands relative to the Fermi energy, with the conduction bands moving upward and the valence bands moving downward. Notably, despite this shift, the HSE06 calculations confirm the nonlinear band dispersion of HXGY without indicating an appreciable gap opening in the band structure.

**Figure 6:**
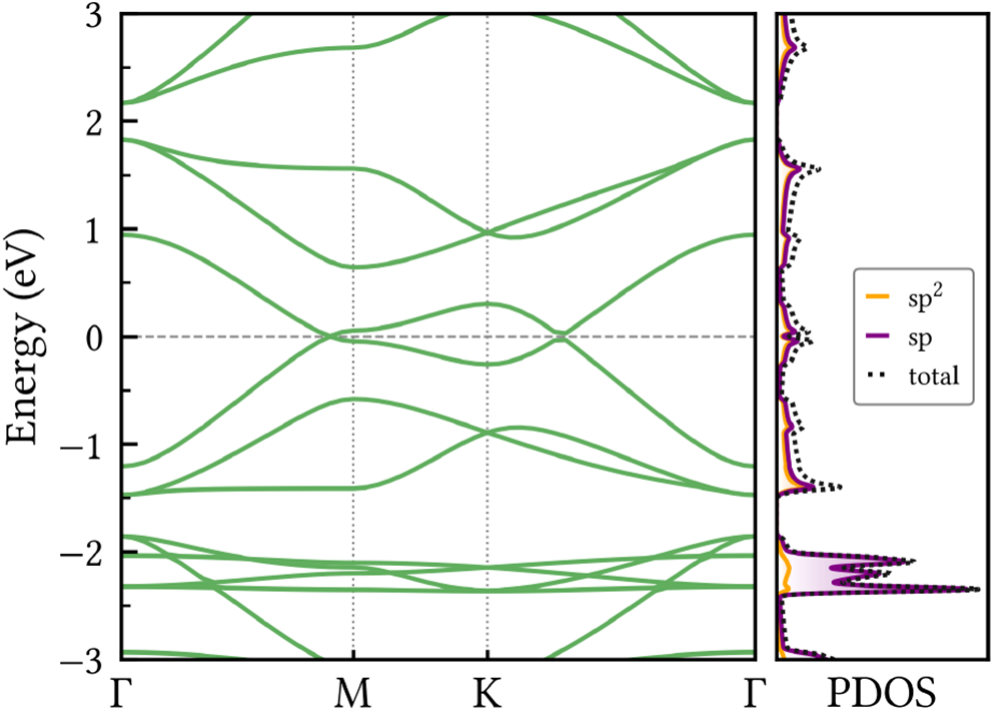
Electronic band structure and projected density of states (PDOS) of HXGY calculated at the HSE06 level. The horizontal gray dashed line indicates the Fermi energy.

#### 1D Nanoribbons

Given that nanoribbon synthesis is often more feasible than that of extended 2D layers for some carbon allotropes, we also investigated quasi-1D finite fragments of HXGY. All nanoribbons considered in this work are hydrogen-passivated at the edges in order to remove dangling-bond states and ensure electronic stability. Hydrogen termination effectively saturates undercoordinated carbon atoms, suppressing spurious edge-localized states near the Fermi level.^46^ While alternative passivating species such as −OH or −F could further tune edge dipoles and electronic states, hydrogen passivation provides a well-defined reference for isolating intrinsic width- and topology-dependent electronic effects.

Figure 7 presents six nanoribbons of different widths, but infinite along the *y*-direction, derived from HXGY and grouped according to their edge topology and increasing width. Panels (a–c) illustrate nanoribbons terminated by an atomic pattern that resembles a zigzag edge. As the ribbon width increases from *n* = 1 to 2, one dodecagonal pore unit is added symmetrically, progressively recovering the periodicity of the planar lattice. Conversely, the nanoribbons in panels (d–f) feature an atomic pattern characteristic of an armchair edge. Once more, the width increases with the addition of one distorted hexagonal pore unit, maintaining topological consistency across the series.

**Figure 7:**
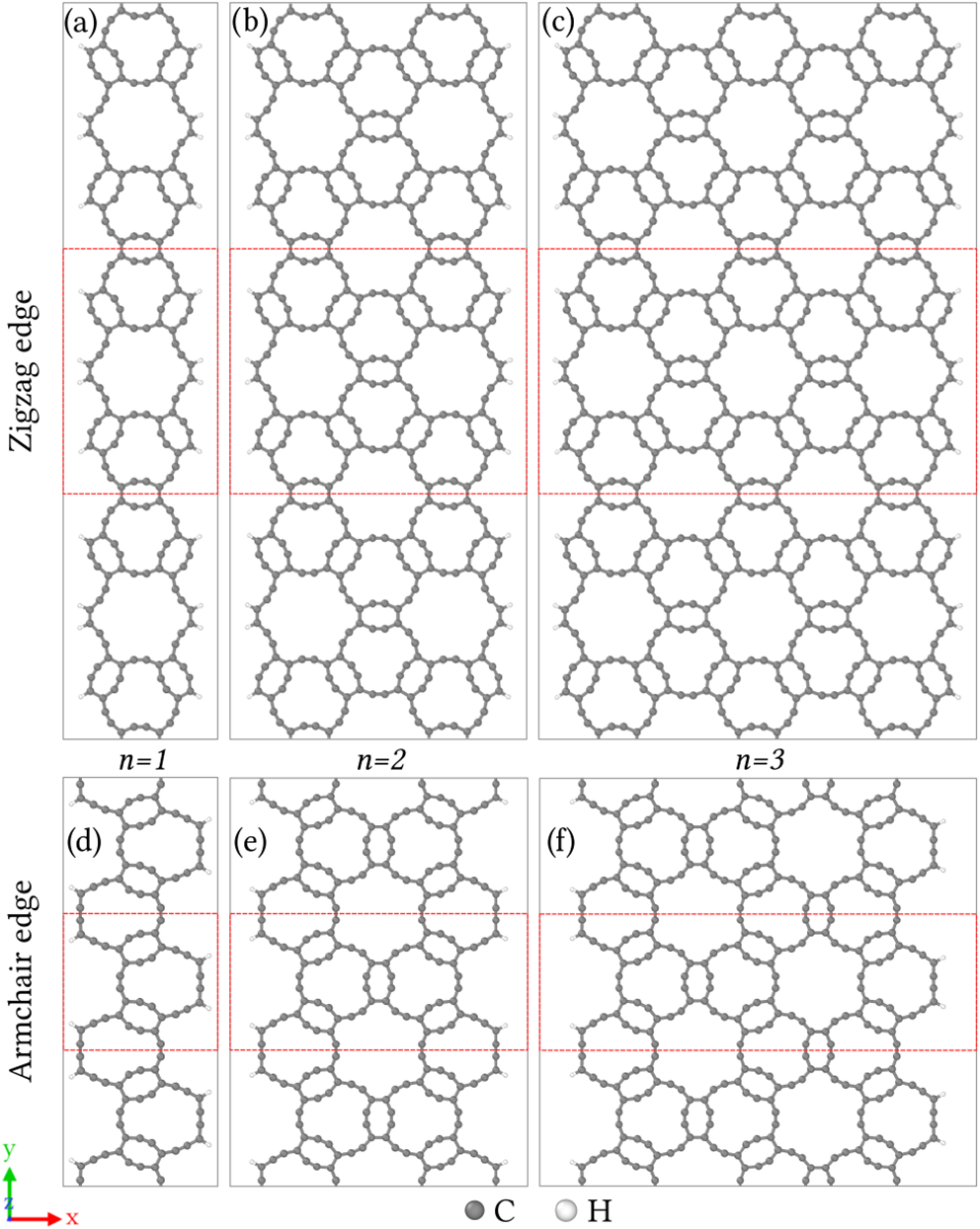
Optimized atomic structures of hydrogen-passivated nanoribbons derived from HXGY with (a–c) zigzag edge type, and (d–f) armchair edge type. The width increases from left to right by adding one dodecagonal or hexagonal pore unit. The periodic is along the *y* axis and red dashed lines represent the unit cells of these systems.

For the two nanoribbon families presented in Figure 7, we conducted full structural relaxation and electronic structure calculations. The resulting geometric parameters and electronic band gaps are compiled in Table 1. Further analysis of the results indicates the lattice parameter along the periodic direction remains nearly constant for a given nanoribbon type, regardless of its width. Additionally, the zigzag nanoribbons exhibit a unit cell with both a lattice parameter and an atom count approximately double those of the armchair nanoribbons. This fundamental difference stems from the distinct crystallographic slicing directions used to define the two ribbon families from their 2D counterpart lattice.

**Table 1:** Geometric Parameters and Electronic Band Gaps for HXGY, Zigzag (Z-HXGYNR), and Armchair (A-HXGYNR) Nanoribbons

		Z-HXGYNR-*n*			A-HXGYNR-*n*		
structural data	HXGY	*n* = 1	*n* = 2	*n* = 3	*n* = 1	*n* = 2	*n* = 3
width (Å)		11.85	25.86	39.91	13.45	25.54	37.76
lattice parameter (Å)	14.05	24.54	24.43	24.40	13.26	13.76	13.86
number of atoms	36	72	144	216	40	76	112
*E*_gap_ (eV)	0	0	0.10	0.04	0.40	0	0

Figure 8 presents the electronic band structure of the six selected nanoribbons derived from HXGY. Panels (a–c) correspond to nanoribbons with zigzag-type edges, while (d–f) correspond to armchair-type ones.

**Figure 8:**
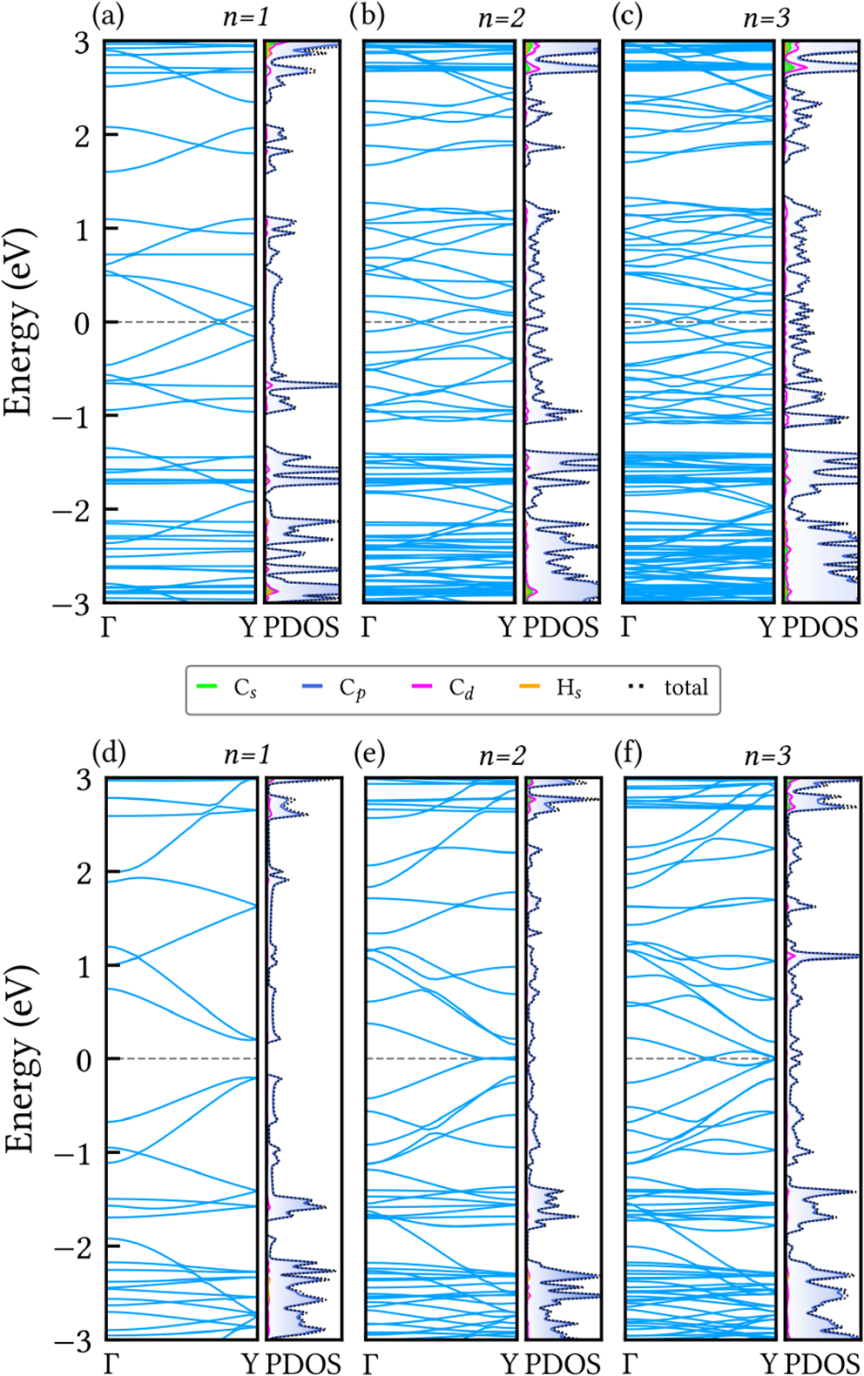
Electronic band structure and projected density of states (PDOS) of width-dependent HXGY nanoribbons with (a–c) zigzag-type edges, and (d–f) armchair-type edges. The horizontal gray dashed line denotes the Fermi level.

The electronic band gaps of HXGY nanoribbons, summarized in Table 1, reveal a notable nonmonotonic dependence on ribbon width, which originates from the interplay between quantum confinement and edge-induced symmetry breaking in narrow ribbons, while increasing the width progressively restores bulk-like band overlap characteristic of the parent 2D lattice. Hydrogen passivation suppresses dangling-bond states, ensuring that the observed semiconductor-to-semimetal transitions are intrinsic rather than defect-driven.

The narrowest zigzag-type nanoribbon (*n* = 1) exhibits a gapless semimetallic character, similar to its planar counterpart. Upon increasing the width to *n* = 2, a true semiconductor phase emerges, characterized by a larger, direct band gap of 0.10 eV and no states near the Fermi level (see Figure 8b). For the widest zigzag-type nanoribbon (*n* = 3), the system returns to a semimetallic state, with a reduced gap of 0.04 eV. In general, the zigzag-type nanoribbons still feature forbidden energy intervals below/above a set of valence/conduction bands, similar to their planar counterpart.

In contrast, the armchair-edged nanoribbons display a markedly different electronic behavior dependence on width. The electronic structure of the narrowest armchair-type nanoribbon (Figure 8d) exhibits a semiconducting character with a direct band gap of 0.40 eV. This large band gap can be attributed to the edge-induced symmetry-breaking and quantum confinement effects inherent to the reduced dimensionality. For *n* = 2, the band gap collapses to zero, indicating a transition to a semimetallic state. This abrupt closure of the gap indicates that at a critical width, the system develops a new electronic configuration where the valence and conduction bands begin to overlap. This semimetallic character is maintained in the widest armchair-type nanoribbon (Figure 8f), which shows the same gapless nature. This width-dependent electronic transition resembles what is observed in armchair graphene nanoribbons.^7^ This semiconductor to semimetal transition demonstrates that the electronic phase of HXGY nanoribbons is tunable with width, a critical property for designing application specific nanoelectronic devices.

PDOS analysis from Figure 8 confirms that for all ribbons studied, the electronic states near the Fermi level are dominated by the carbon p orbitals. Consequently, the carbon s and d orbitals remain largely inactive near the Fermi level, as well as hydrogen *s* orbitals.

### Optical Properties

The optical properties of HXGY, including absorption, reflectivity, and refractive index, are summarized in Figure 9. The overall optical response is nearly isotropic in the plane, a desirable trait for applications requiring polarization independence. To address the known underestimation of optical excitation energies by the PBE functional, we also calculated the optical spectra using the HSE06 hybrid functional for a more accurate description. For all optical coefficients depicted in Figure 9, the HSE06 spectra is slightly shifted to higher energies relative to PBE. This shift is consistent with the known tendency of hybrid functionals to increase the band gap and partially correct the underestimated transition energies of semilocal functionals.^47^

**Figure 9:**
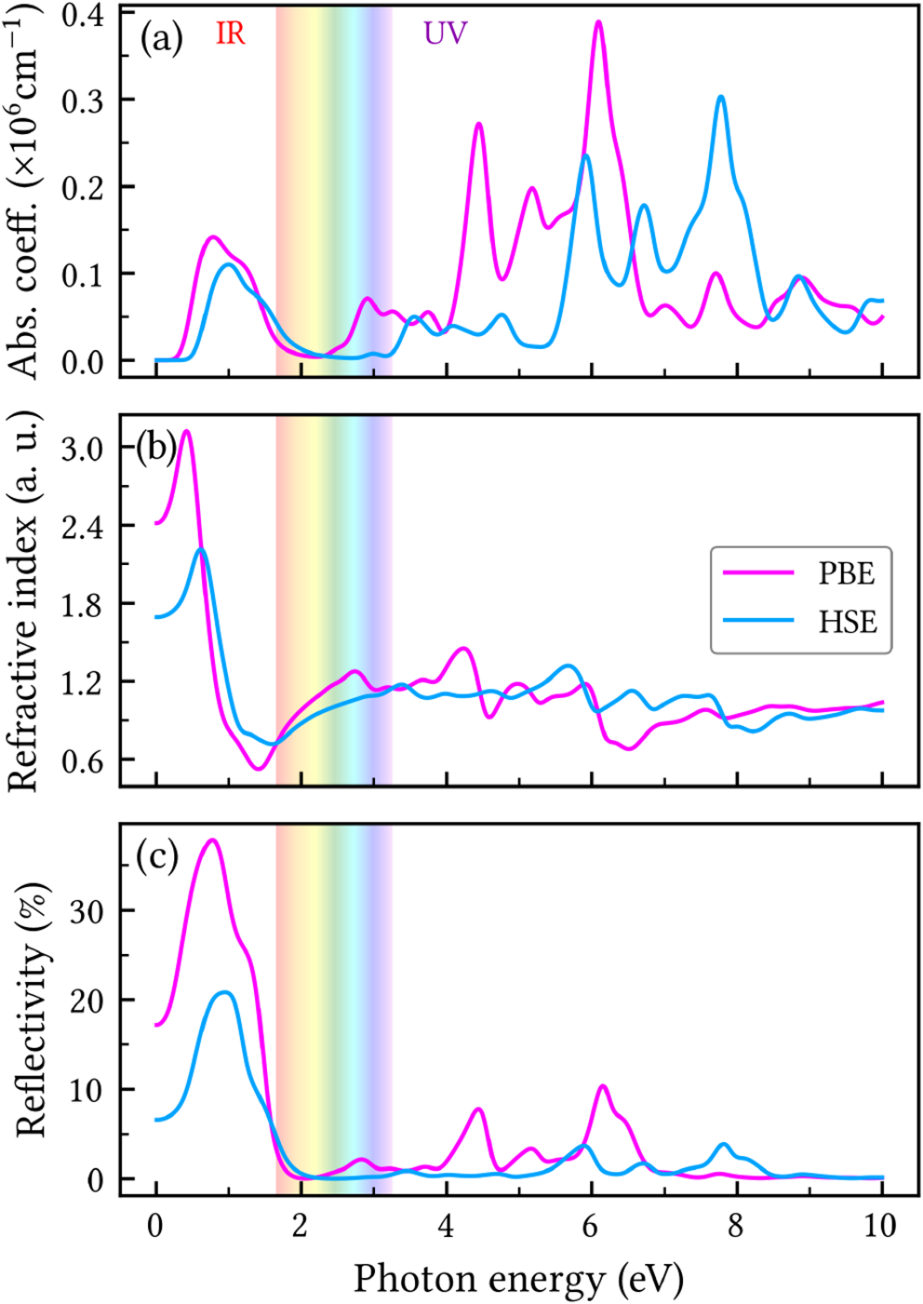
In-plane optical coefficients of HXGY: (a) absorption coefficient, (b) refractive index, and (c) reflectivity as a function of photon energy. The visible light range is indicated by the colored region.

As evidenced by panel (a) of Figure 9, HXGY exhibits strong absorption in the ultraviolet (UV) region. The absorption coefficient reaches values near 0.4 × 10^6^ cm^–1^ (0.3 × 10^6^ cm^–1^) at PBE (HSE06) level. In contrast, the absorption in the infrared (IR) range is more moderate, while it diminishes significantly within the visible region. Compared to α-, β-, and γ-GY, the absorption spectrum of HXGY is characterized by more intensive peaks in the investigated energy range.^15,48^ Specifically, stronger absorption activity in the UV region is also observed in α-, and γ-GY, which contrasts sharply with β-GY, which demonstrates significantly stronger absorption in the IR range.^15^

The visible transparency of HXGY is a key finding and can be directly understood in terms of its electronic band structure and optical response. In particular, the imaginary part of the dielectric function, which governs the interband contribution to optical absorption, provides direct insight into the availability and strength of vertical valence-to-conduction transitions. In the visible photon-energy range (approximately 1.6–3.1 eV), both the absorption coefficient and the reflectivity are very small. This behavior indicates that interband transitions are not strictly absent, but rather strongly suppressed, as the relevant transitions in this energy window are characterized by a low joint density of states and/or small optical dipole matrix elements. As a result, HXGY exhibits an effectively transparent response in the visible spectrum. In contrast, pronounced absorption features emerge only at higher photon energies in the ultraviolet region, where interband transitions become significantly more probable. At low photon energies in the infrared, the enhanced reflectivity is consistent with a free-carrier, Drude-like contribution to the optical response.

The refractive index presented in panel (b) of Figure 9 exhibits a sharp peak in the vicinity of 0.5 eV, followed by a steep decline in the near-IR region. A subsequent increase is observed throughout the visible range, before stabilizing with minor oscillations around a value of 0.9 across the remaining spectrum.

In the bottom panel of Figure 9, the reflectivity is characterized by a broader, prominent peak near 0.8 eV, which can be attributed to the pronounced effect of free carriers at low frequencies. At higher energies (visible/UV), HXGY becomes highly transparent, as indicated by its negligible reflectivity in these regions. Unlike α-, β-, and γ-GY, which are highly reflective in the visible range,^15,48^ HXGY exhibits visible-light transparency. This visible transparency is particularly desirable for coating and filtering applications that require minimal optical loss.

### Vibrational Properties

Figure 10 shows the detailed simulated vibrational fingerprint of HXGY. The Raman spectrum in panel (a) is characterized by sharp and well-separated peaks. The three most intense modes can be identified in the spectrum at 988, 1048, and 2059 cm^–1^. The highest-frequency peak at 2059 cm^–1^ is characteristic of symmetric in-plane stretching of acetylenic linkages within the rectangular rings. This is illustrated in the inset and is a feature commonly observed in the GY family.^49^ The lower-frequency Raman peaks are associated with in-plane symmetric and asymmetric vibrations involving sp^2^-hybridized carbon atoms in the rectangular rings.

**Figure 10:**
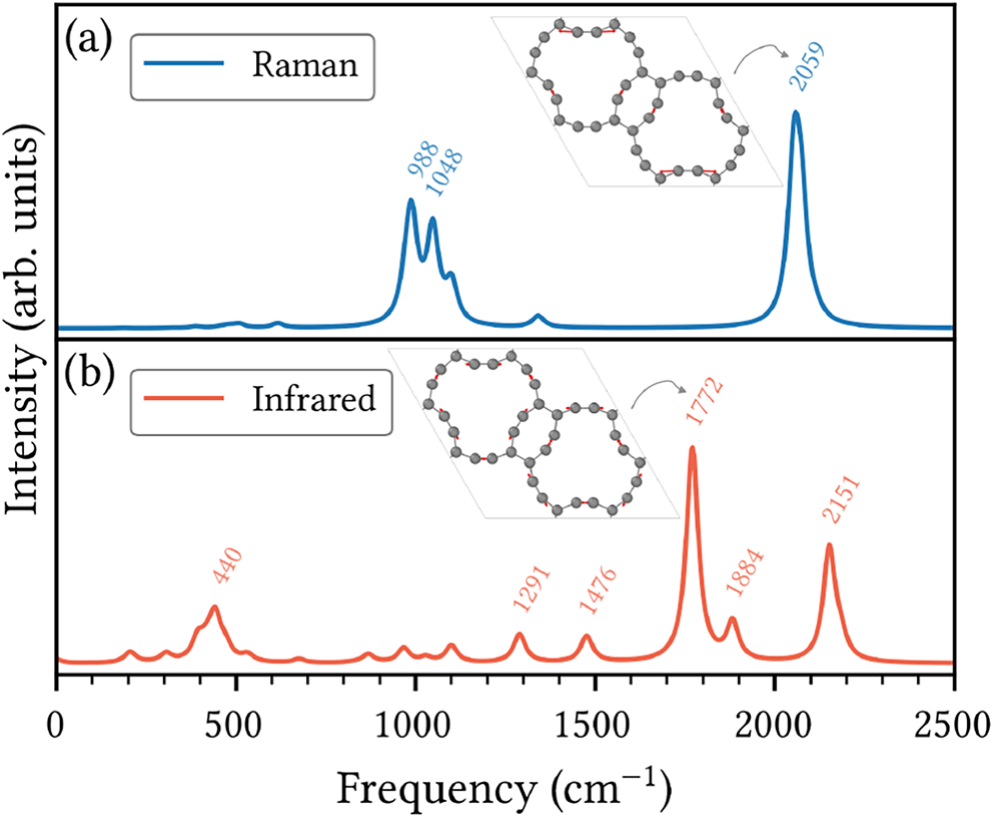
Simulated Raman and infrared spectra of HXGY with labeled peak frequencies (in cm^–1^). The insets display atomic displacement vectors for the most intense modes, where red arrows indicate the direction and relative magnitude of atomic motion.

The infrared (IR) spectrum in the bottom panel of Figure 9 reveals several active modes, with prominent peaks near 440, 1291, 1476, 1772, 1884, and 2151 cm^–1^. The lowest-frequency mode at 440 cm^–1^ corresponds to bending vibrations of sp-hybridized carbon atoms in the rectangular rings. The most intense IR peak, at 1772 cm^–1^, is assigned to stretching vibrations of the acetylenic linkages, as evidenced by the inset.

The distinct and well-separated vibrational fingerprints provide a unique spectral signature that would facilitate the HXGY experimental detection through Raman and infrared spectroscopy.

## Summary and Conclusions

In this work, we conducted a comprehensive characterization of Hexa-graphyne (HXGY), a two-dimensional carbon allotrope composed of distorted hexagonal and rectangular rings featuring sp- and sp^2^-hybridized carbon atoms. First-principles calculations confirm its stability, as evidenced by the absence of imaginary frequencies in the phonon dispersion and by the preservation of structural integrity at temperatures up to at least 1000 K in *ab initio* molecular dynamics simulations.

The present study focuses on monolayer HXGY and its one-dimensional nanoribbon derivatives. The stacking behavior of bilayer or multilayer HXGY structures, substrate-induced effects, as well as chemical reactivity and bond stability under oxidative or functional environments, although highly relevant from an experimental perspective, were not investigated here and therefore constitute natural and important directions for future studies.

Electronic structure calculations confirm the semimetallic nature of monolayer HXGY. In contrast, nanoribbons derived from this material exhibit distinct electronic behaviors depending on their width and edge termination. From a mechanical standpoint, HXGY is a highly compliant and isotropic material, exhibiting a Young’s modulus approximately 13 times lower and a Poisson’s ratio nearly four times greater than those of graphene. Its optical response is also isotropic and characterized by strong ultraviolet absorption, high infrared reflectivity, and pronounced transparency in the visible-light range. Simulated Raman and infrared spectra display sharp and well-separated peaks, with the most prominent modes unambiguously assigned to the stretching vibrations of the acetylenic linkages, providing a clear vibrational fingerprint for experimental identification.

Taken together, these properties, particularly the combination of strong ultraviolet absorption and visible-light transparency, suggest that HXGY may be a promising candidate for transparent UV-protective coatings, selective photodetectors, and related optoelectronic applications. Although, quantitative performance comparisons with established materials remain to be addressed.

## Supplementary Material



## Notes

Crystallographic Information File (CIF) for hexa-graphyne (CIF)
